# Higher *Fusarium* Toxin Accumulation in Grain of Winter Triticale Lines Inoculated with *Fusarium culmorum* as Compared with Wheat †

**DOI:** 10.3390/toxins8100301

**Published:** 2016-10-18

**Authors:** Tomasz Góral, Halina Wiśniewska, Piotr Ochodzki, Dorota Walentyn-Góral

**Affiliations:** 1 Department of Plant Pathology, Plant Breeding and Acclimatization Institute-National Research Institute, Radzików, 05-870 Blonie, Poland; p.ochodzki@ihar.edu.pl (P.O.); d.walentyn-goral@ihar.edu.pl (D.W.-G.); 2 Institute of Plant Genetics, Polish Academy of Sciences, 34 Strzeszynska str., 60-479 Poznan, Poland; hwis@igr.poznan.pl

**Keywords:** *Fusarium*, triticale, wheat, deoxynivalenol, zearalenone

## Abstract

Resistance to *Fusarium* head blight in 32 winter triticale and 34 winter wheat accessions was evaluated. Triticale and wheat were sown in field experiments in two locations. At the time of flowering, heads were inoculated with three *Fusarium culmorum* isolates. *Fusarium* head blight index was scored and after the harvest percentage of *Fusarium* damaged kernels was assessed. Grain was analysed for type B trichothecenes (deoxynivalenol and derivatives, nivalenol) and zearalenone (ZEN) content. The average *Fusarium* head blight indexes were 28.0% for wheat and 19.2% for triticale accessions. The percentage of *Fusarium* damaged kernels was also higher for wheat and came to 55.6%, while for triticale this figure was 40.2%. The average content of deoxynivalenol (DON) for wheat amounted to 11.65 mg/kg and was lower than the result for triticale which was 14.12 mg/kg. The average contents of nivalenol were similar in both cereals: 4.13 mg/kg and 5.19 mg/kg for wheat and triticale respectively. Considerable amounts of DON derivatives in the cereals were also detected. The ZEN content in the grain was 0.60 mg/kg for wheat and 0.66 mg/kg for triticale. Relationships between *Fusarium* head blight index, *Fusarium* damaged kernels and mycotoxin contents were statistically significant for wheat and mostly insignificant for triticale. Triticale proved to have less infected heads and kernels than wheat. However, the content of type B trichothecenes was higher in triticale grain than in wheat grain.

## 1. Introduction

Hexaploid triticale (× *Triticosecale* Wittmack) is an artificially created cereal, resulting from the crossing of wheat (*Triticum* sp. Linnaeus) and rye (*Secale cereale* Linnaeus) [1]. The maternal parents were bread wheat (*T. aestivum* Linnaeus) or durum wheat (*T. durum* Desfontaines). Stable hexaploid types of triticale were obtained directly from crosses with durum wheat or crosses between hexaploid and octoploid forms which resulted in secondary triticale populations. Hexaploid triticale includes the A and B genomes of *Triticum* and R of *S. cereale*.

There is great interest in this cereal in Poland, due to the considerable share of poor and acid soils in the overall area of arable land. Advantages of triticale are the high yielding capacity and good quality grains intended for feed, characterised by a high content of proteins of the favorable composition of aminoacids and a high digestibility coefficient [2].

Triticale, as an entirely synthetic genus, has low genetic variation due to lack of transition through the process of evolution. In addition, the selection of donors of triticale subgenomes (wheat, rye), led by breeders contributes to the reduction of the genetic variability of varieties including limited diversity of traits responsible for the resistance to the biotic and abiotic stresses.

The development of breeding techniques, such as the production of doubled haploids, have strongly accelerated the process of breeding by introducing pure lines, but have also narrowed the genetic variation. The lack of an intentional selection pressures towards resistance to stresses and the conditions of the cultivation of plants used in breeding stations have caused modern varieties to be poorly adapted to biotic and abiotic stresses [3,4,5].

*Fusarium* head blight (FHB) is a disease of cereals caused by pathogenic fungi of the genus *Fusarium* [6,7]. Pathogenesis of FHB is complex. There are several types of resistance to FHB: resistance to initial infection (type I); resistance to *Fusarium* spread along the rachis (type II), resistance to kernel infection (type III); tolerance against FHB or trichothecenes (type IV); and resistance to accumulation of trichothecenes (type V) [8,9]. Resistance to FHB is complex and is a multigenic feature. Presence or a few or more quantitative trait loci (QTL) for resistance provides sufficient field resistance of plants to diseases [10,11]. Each of the genes (QTLs) determines a relatively small quantitative effect, however, combining their effects increases disease resistance levels [12,13]. Expression of quantitative resistance (e.g., to FHB) is substantially modified by the environmental conditions. High air humidity, wind and rain are the factors promoting the development of FHB [14].

*Fusarium* head blight may lead to a reduction in yield of grain. However, the concern related to FHB is contamination of grain with mycotoxins such as deoxynivalenol (DON), nivalenol (NIV), zearalenone (ZEN), which are extremely stable; they are not metabolized and are harmful to humans and animals [15,16]. Contamination of grain with mycotoxins is observed even when there is no reduction in the yield [17].

Fungi of the genus *Fusarium* produce several toxins that can be strongly or chronically toxic to both humans and animals, depending on the type of toxin and the amount of consumed food or feed. The consumption of food or feed contaminated with mycotoxins causes a variety of diseases in humans and animals known generally as mycotoxicoses. Trichotecenes (DON, NIV) have strong toxic effects, such as skin irritation, vomiting, diarrhea, weakness, decreased appetite, hemorrhages, neurological disorders, miscarriages and may even lead to death [18]. Zearalenone causes hyperestrogenic syndromes in pigs and can lead to disrupted conception. Some types of *Fusarium* mycotoxins (zearalenone, fumonisins) are associated with a growing number of cancers in humans [15]. Taking into consideration the facts above, the content of mycotoxins in grain cereals requires control and should be reduced. The limits of *Fusarium* toxins in food are regulated by Commission Regulation No. 1126/2007 of 28 September 2007 amending Regulation (EC) No. 1881/2006, which sets maximum levels for certain contaminants in foodstuffs with regard to *Fusarium* toxins in maize and maize products. Limits for animal feed are less restrictive and are regulated by Commission Recommendation No. 2006/576/EC on the presence of deoxynivalenol, zearalenone, ochratoxin A, T-2 and HT-2 and fumonisins in products intended for animal feed. However, for pig feed, standards are much tougher, the latter standards are particularly important for triticale grain, which is used mostly for feed, to a large extent in feeding pigs.

*Fusarium* head blight of wheat has been studied for many years in different countries, and there is much data on this cereal. However, the resistance of triticale to this disease is not as well known. *Fusarium* head blight is the most damaging to bread wheat and durum wheat. In comparison to other cereals, durum wheat is the most susceptible to this disease. Most of the published papers on triticale show that in terms of FHB resistance, it is more resistant than bread wheat and less resistant than rye [19,20,21,22,23]. However, there are results available showing that susceptibility of triticale to *Fusarium* head blight may be higher and even equal to that of wheat and considerable amounts of *Fusarium* toxins can be accumulated in triticale grain [24,25,26]. There are a small number of studies of the topic, due to the fact that triticale is a relatively new species and it is grown in much smaller areas of the world than wheat. However, in Poland, acreage of triticale is about 50% of the wheat acreage. Hence, it is important to obtain knowledge about the resistance of this cereal to *Fusarium* head blight, which can be used in breeding programs.

Many new varieties of triticale are susceptible to FHB, at a level similar to wheat. There are reports that in the triticale grain the amount of accumulated mycotoxin may be similar or even higher than that found in wheat, despite a lower severity of disease on spikes and kernels. So far there is no explanation for this phenomenon. Canadian researchers suggest that increased accumulation of *Fusarium* toxins results from higher susceptibility of triticale pericarp to *Fusarium* damage [26,27]. Using scanning electron microscope, we have observed that triticale kernels were more damaged by *Fusarium* in comparison to wheat [28,29]. The other reason for the vulnerability of part of the genetic pool of triticale (mainly spring), may be that the original forms were obtained from crosses of rye and durum wheat, which have a very high susceptibility to FHB, e.g., early Canadian cultivar “Rosner” [30] or “Armadillo” developed in CIMMYT (International Maize and Wheat Improvement Center, Mexico) [31]. In contrast, the European winter triticale genetic pool was developed in Hungary and Poland from secondary triticales obtained by hexaploid x octoploid crosses which included the genome of bread wheat, which is more resistant to FHB [1].

The main source of resistance is the *Fhb1* gene located in the short arm of chromosome 3B. This is a major gene for resistance to FHB, present in Chinese spring wheat variety “Sumai 3”, which is the main source of resistance to this disease. Additionally, another gene, *Fhb2*, located on the short arm of chromosome 6B, is well characterised. It has, however, much less of an impact on the total resistance to FHB [32].

In addition, in triticale, as an artificial form, the erosion of the genetic base of varieties is at risk if no crosses of parental species are made. This can lead to a decrease in resistance of this species for pathogens, including *Fusarium* spp. There are attempts being made to broaden the variation of triticale by introducing genes from, for example, *Triticum monococcum* and *Aegilops* species and from the highly resistant bread wheat “Sumai 3” [33,34,35,36].

This study evaluated winter triticale and winter wheat breeding lines for their reactions to FHB after inoculations with *Fusarium culmorum* in field trials. Data were analysed to reveal if there was a statistical difference between triticale and wheat in head infection, kernel damage and accumulation of *Fusarium* toxins (type B trichothecenes, zearalenone) in grain.

## 2. Results

Triticale flowered in both locations about 7–10 days earlier than wheat (Supplementary Materials Table S1). Initiation of flowering of both cereals was about 5 days earlier in Cerekwica than in Radzików. Weather conditions in Cerekwica during inoculations of triticale accession were unfavourable, because of high air temperatures and lack of precipitation (Supplementary Materials Figure S1). However, in Cerekwica mist irrigation was applied post-inoculation. In Radzików, weather during triticale inoculation was more favourable for *Fusarium* infections because of daily occurrence of rainfall. However, air temperature was low at the beginning of June (10.1 °C on 2 June). Weather conditions during inoculation of wheat in Cerekwica were more favourable than for triticale, because of occurrence of rainfall (Supplementary Materials Figure S1). The temperature was low only during the first three days of inoculations (10.2 °C on 5 June). In Radzików, weather conditions were even better because of the high temperature accompanied by frequent rainfalls.

Post-inoculation conditions in both locations were favorable for FHB development and mycotoxin production (Supplementary Materials Table S2). Weather, until the end of harvest, was rainy and with medium to high average daily air temperatures.

The average severity of FHB in 32 winter triticale lines was similar in both locations and amounted to 19.8% in Radzików and 18.7% in Cerekwica. The range of reactions was from 13.7% to 32.0% in Radzików and from 4.8% to 40.0% in Cerekwica. The proportion of *Fusarium* damaged kernels was higher in Cerekwica (53.7%) than in Radzików (26.8%). The range of reaction was from 9.4% to 45.0% in Radzików and from 27.5% to 68.9% in Cerekwica.

Average DON content in grain in Radzików amounted to 8.69 mg/kg and was lower than in the second location, which had DON content of 19.54 mg/kg. DON content ranged from 7.36 to 37.39 in Cerekwica, and from 4.41 to 17.43 mg/kg in Radzików. In grain samples from Cerekwica there were also high quantities of NIV. The average content was 10.05 mg/kg. In Radzików NIV content was low—0.32 mg/kg on average. There was also a significant amount of DON derivatives (3-acetyldeoxynivalenol [3AcDON], 15-acetyldeoxynivalenol [15AcDON]) in triticale grain in both locations (1.82 mg/kg of 3AcDON and 1.91 mg/kg of 15AcDON, on average). The content of ZEN in triticale grain from Cerekwica was very high and amounted to 1.12 mg/kg (0.51–2.75 mg/kg), while in the grain from Radzików this figure was 6 times lower at 0.20 mg/kg (0.03–1.08 mg/kg). Average values from the locations for 32 winter triticale accessions are presented in Supplementary Materials Table S3.

In both locations, parallel inoculation experiments with 34 winter wheat lines were carried out. The average severity of FHB in winter wheat lines was higher in Radzików than in Cerekwica and amounted to 34.2% and 21.8%, respectively. The range of reactions was from 22.5% to 55.0% in Radzików and from 8.3% to 49.5% in Cerekwica. The proportion of *Fusarium* damaged kernels was higher in Cerekwica (83.3%) than in Radzików (28.1%). The range of reaction was from 13.7% to 49.5% in Radzików and from 55.1% to 95.3% in Cerekwica.

Average content of DON in grain in Radzików amounted to 8.70 mg/kg and was lower than in the second location, which had a content of 14.87 mg/kg. DON content ranged from 7.36 to 37.39 mg/kg in Cerekwica, and from 4.41 to 17.43 mg/kg in Radzików. In grain samples from Cerekwica there were also high quantities of NIV. The average content was 7.88 mg/kg. In Radzików NIV content was low—0.39 mg/kg on average. There was also a significant amount of derivatives of DON in wheat grain in both locations (2.00 mg/kg of 3AcDON and 1.47 mg/kg of 15AcDON, on average). The content of ZEN in wheat grain from Cerekwica was very high and amounted to 1.03 mg/kg (0.41–3.15 mg/kg), while in the grain from Radzików this figure was six times lower—0.17 mg/kg (0.03–0.83 mg/kg). Average values from the locations for 32 winter wheat accessions are presented in Supplementary Materials Table S4.

**Figure 1:**
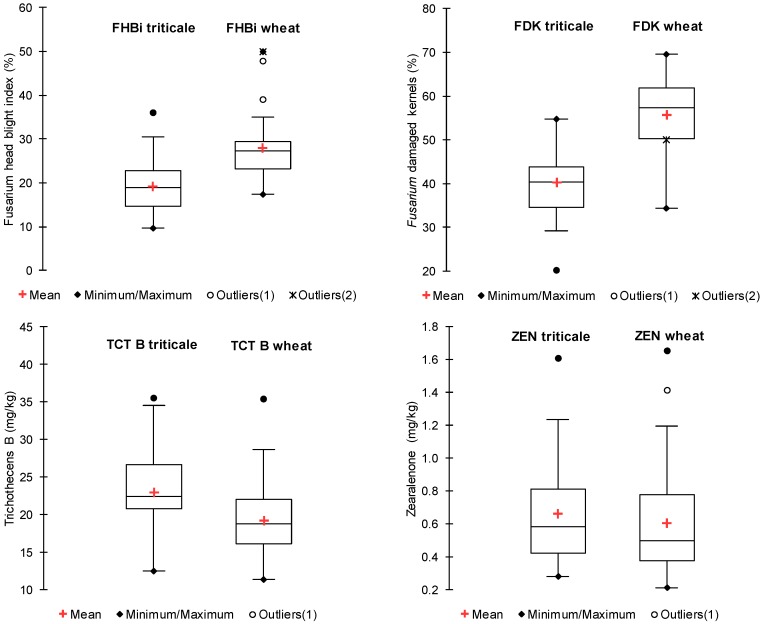
*Fusarium* head blight index (FHBi), *Fusarium* damaged kernels (FDK) proportion and accumulation type B trichothecenes (TCT B) and zearalenone (ZEN) in grain of triticale and wheat inoculated with *F. culmorum*. Boxes represent first quartile, median, and third quartile. Whiskers show lower and upper limits.

Triticale lines were less *Fusarium*-infected than wheat with regard to heads and kernels (Figure 1). The differences were statistically significant (Table 1). However, the content of all trichothecene toxins was higher in triticale grain than in wheat grain (Figure 1). Statistically significantly higher was the content of DON, 15AcDON and NIV (Table 1). ZEN content was higher in triticale grain; however, the difference was not statistically significant. Variability in wheat and triticale was similar for FHB index, *Fusarium* damaged kernels (FDK), type B trichothecenes and ZEN (Figure 1). No significant differences were found between variances for FHB index, FDK, sum of type B trichothecenes and ZEN contents. However, for individual trichothecenes (DON, 15AcDON and NIV) variances were 2 times higher in triticale than in wheat.

**Figure 2:**
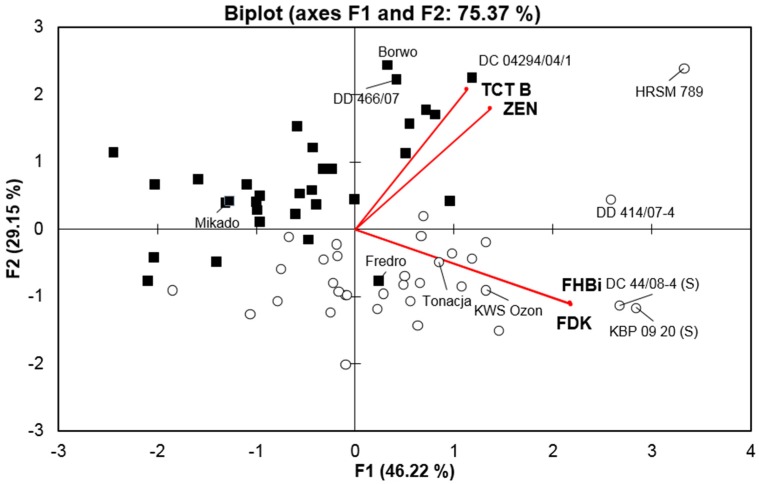
Biplot of the principal component analysis of *Fusarium* head blight index (FHBi), *Fusarium* damaged kernels (FDK), type B trichothecenes (TCT B) and zearalenone (ZEN) accumulation in grain for 32 lines of winter triticale (squares) and 34 lines of winter wheat (circles) inoculated with *F. culmorum* in Radzików and Cerekwica. (S): susceptible checks of winter wheat.

In the principal component analysis of FHB index, *Fusarium* damaged kernels and concentration of type B trichothecenes and zearalenone for both cereals showed a clear division into two groups (Figure 2). In the first group most of triticale lines were grouped (except “Fredro” cv.), which showed lower head and kernel infection and higher toxin accumulation. Wheat lines of more infected heads and kernels and lower accumulation of *Fusarium* toxins formed the second group. The exceptions were two wheat lines (“DD 414/07-4”, “HRSM 789”) which were highly infected and accumulated high amount of toxins in the grain. Two susceptible lines “DC 44/08-4 (S)” and “KBP 09 20 (S)” showed medium toxin accumulation lower than some less infected triticale lines e.g., “DC 04294/04/2”, “MAH 33881-1/3”, “DC 04294/04/1”, “CM 9/10”, “Borwo”, “DD 466/07”.

In triticale average FHB index correlated significantly with FDK, and concentrations of DON, 3AcDON and sum of type B trichothecenes (Table 2). Coefficients, however, were low. On the other hand, FDK proportion correlated significantly with content of NIV, ZEN and sum of type B trichothecenes. Coefficients were higher than for FHB index. The accumulation of type B trichothecenes and ZEN also correlated highly significantly. We found significant, negative correlation of DON and NIV concentrations.

In wheat, average FHB index correlated significantly with FDK proportion and mycotoxin concentrations, except 15AcDON and NIV (Table 2). FDK also correlated significantly with mycotoxins, except NIV and ZEN content. The accumulation of type B trichothecenes and ZEN did not correlate significantly. Coefficients of correlations of FHB index versus FDK, DON, TCT B were higher in wheat (0.538–0.604) than in triticale (0.357–0.408).

## 3. Discussion

The main finding of the present research is that in certain conditions triticale can accumulate in grain more type B trichothecene toxins than wheat. Accumulation of large amounts of DON in triticale grain was found previously in different studies [19,20,37,38]. Published results, however, were highly dependent on the triticale population was used in the experiments. Miedaner et al. [19] and Góral and Ochodzki [38] found that German and/or Polish triticale cultivars were more resistant to accumulation of DON in the grain than wheat. In contrast, Polish [37] and German breeding lines [20] accumulated high amounts of DON in grain exceeding that reported for wheat [39].

A possible explanation of the high DON content in triticale grain could be the different structure of triticale kernel as compared to wheat kernel. Triticale kernels are, on average, softer than wheat and tend to have higher water uptake rate than wheat kernels [40,41]. Canadian researchers observed that triticale kernels are (as compared to wheat), more susceptible to damage caused by *Fusarium* [25,26,27]. The pericarp of triticale is fragile to *Fusarium* infection/toxin contamination, which results in greater kernel destruction than in wheat [29]. However, it should be noted that proportion of *Fusarium* damaged kernels in triticale is mostly lower than in wheat (Figure 1) [21,42]. This is the effect of lower head infection observed in triticale as compared to wheat [20,21,43,44,45,46].

The other factor influencing observed FHB severity in triticale and wheat heads could be differences in flowering time between the two cereals. Triticale generally flowers earlier than wheat. In our research the difference was 7–10 days. Weather conditions have significant effect on infection of heads with *Fusarium* and later development of FHB symptoms [14]. Weather also strongly modifies *Fusarium* mycotoxins production under a field conditions [47,48]. Similar head infection symptoms or kernel damage under different environmental conditions does not result in the same DON levels. Application of mist irrigation post-inoculation can partially remove the effect of unfavourable weather conditions [49,50]. In present research mist irrigation was applied in one experimental location—Cerekwica. However, under field conditions, we cannot control the air temperature, which also affects the development of FHB symptoms and mycotoxin production.

Resistance to FHB in wheat and in triticale is described by different types controlled, partially, by different QTLs [32,51]. Type IV refers to the resistance to DON accumulation by inhibition of toxin synthesis or chemical modification of DON [9,52]. The latter is a glycosylation process which ends with production of DON-glucoside, showing no toxicity to plant cells [53]. DON-glucoside and other so called “masked mycotoxins” were detected in different cereal species, usually at up to 20% of unconjugated DON [54]. Rassmussen et al. [55] studied the proportion of this form of DON in different cereal grains and found the highest relative content of deoxynivalenol-3-β-d-glucoside (up to 37% of DON) in triticale. Average concentrations of DON in grain of studied wheat and triticale cultivars were similar and amounted to 6.54 mg/kg and 6.91 mg/kg, respectively. It should be noted that DON content in triticale was slightly higher. These results and results of our research are unfavorable for triticale. Masked mycotoxins can be digested back to their toxic form [54], hence toxic potential of infected triticale grain may be even higher.

We have analysed relationships of FHB resistance components (FHB index, FDK, mycotoxins) in triticale and wheat. In both cereals FHB index correlated significantly with kernel damage and DON and type B trichothecenes accumulation. However, coefficients for wheat were higher and highly significant.

As compared to wheat, in triticale lower coefficients of correlations between FHB symptoms on heads and kernel damage and mycotoxins have been reported. One of the reasons could be more difficult FHB scoring in triticale than in wheat [20,56,57]. As Miedaner et al. [20] stated, this is because of the widely varying colors of triticale heads and awns and the differing shape of its heads. The same conclusions were drawn by Oettler and Wahle [57]. They stated that triticale breeding materials showed significant differences in spike type, awn length and color, or glume color. This made make it difficult in individual genotypes to differentiate clearly visually between bleached spikelets because of infection or bleaching owing to the ripening process. Miedaner et al. [20] found that FDK rating had a higher correlation to DON content than FHB rating and was similar to that for wheat. In our research we found no correlation between FDK and DON in triticale; however, coefficient of correlation with sum of trichothecenes was significant and only slightly lower than that for wheat.

With regard to relationships between mycotoxins, we found high correlations of DON vs. DON acetyl derivatives. It is obvious as 3AcDON or 15AcDON are metabolized to DON by removing of acetyl units by wheat or fungal esterases (deacetylases) [58]. The deacetylation to DON by the fungus occurs slowly, so that during the *Fusarium* infection process the plant is most likely confronted primarily with either 15-ADON or 3-ADON [59]. The non-acetylated DON is next accumulated in wheat grain at amounts much larger than acetylated forms [60].

We found a lack of correlation between NIV and DON (3AcDON, 15AcDON) accumulation for wheat and significant negative correlation DON vs. NIV in triticale. Trichothecenes (DON, 3AcDON, 15AcDON) are *Fusarium* virulence factors inhibiting plant defense response in advance of the invading intercellular hyphae [61]. Lack of trichothecene synthesis capacity of *Fusarium graminearum* resulted in susceptible plants being able to slow down or even block *Fusarium* spread which remained restricted to the infected florets [62]. *F. graminearum* with disrupted *Tri5* (encoding trichodiene synthase) gene produced much fewer symptoms than wild-type isolates [63]. It was found that also NIV is a FHB virulence factor and causes similar symptoms on wheat heads to those produced by DON [64]. However, differences in the detoxification mechanism of both toxins were observed. Lemmens et al. [64] postulated that the mechanism of resistance of wheat to NIV is different from the one described for DON. In *Fusarium* species able to produce both DON and NIV (*F. culmorum*, *F. graminearum*) these toxins are produced by different chemotypes. Yoshida et al. [65] inoculated wheat with mixture of DON and NIV isolates of *F. graminearum*. They found similar kinetics of production of both toxins. However, results showed different DON:NIV ratio depending on genotype. This was observed in our study and resulted in low or negative correlation coefficients. Different DON:NIV ratios could be the effect of competition between different isolates [66]. Co-inoculations can decrease toxin production. The proportion of toxins in individual genotype in specific experimental conditions can be the effect of an isolate-specific response.

Concentration of zearalenone in triticale grain correlated significantly with type B trichothecenes content. However, we found some lines with low or moderate trichothecene content and high amount of ZEN in grain. In wheat, no correlation ZEN vs. trichothecenes was observed, only a significant correlation with NIV content. We found eight lines with high amounts of ZEN in grain, but showing different trichothecene contents. In contrast to DON, zearalenone was not specified as *Fusarium* virulence factor. It is produced later than DON (or trichothecenes) during pathogenesis [67] or in higher amounts late in the growing season [68,69]. ZEN concentration is greatly affected by the weather conditions late in the season [50]. Late rainfall significantly increased ZEN amount accumulated in wheat grain.

## 4. Conclusions

Winter triticale accessions showed weaker *Fusarium* head blight symptoms (head infection, proportion of damaged kernels) as compared to winter wheat accessions. However, accumulation of *Fusarium* toxins in triticale grain was significantly higher than in wheat grain for type B trichothecenes and there was no significant difference for zearalenone between two cereal species.

The results showed that there is a threat of contamination of triticale grain with mycotoxins despite lower severity of *Fusarium* head blight symptoms. It indicates that in *Fusarium* head blight resistance selection some other resistance components should be scored to avoid genotypes combining low disease symptoms with high toxin accumulation.

## 5. Materials and Methods

The resistance to *Fusarium* head blight was studied with respect to 29 winter triticale lines, and three cultivars: “Borwo”, “Fredro”, and “Mikado”, as well as 32 winter wheat lines and cultivars ”KWS Ozon” and “Tonacja”. All lines originated from Polish breeding companies and were selected from the large set of breeding lines based on low head infection in two environments (data not shown).

The accessions were sown in the field in Cerekwica near Poznań (GPS coordinates 52.522579, 16.688624) and in Radzików near Warsaw (GPS coordinates 52.212612, 20.633111). Both experiments were established as a randomized block design. Wheat cultivars were sown in 1 m^2^ plots in three replicates/blocks.

The infective material was a mixture of three isolates of *Fusarium culmorum*, producing deoxynivalenol (KF846, DON chemotype), nivalenol (KF350, NIV chemotype) and zearalenone (ZFR 112, DON chemotype) [42]. Isolates were incubated on autoclaved wheat kernels in glass flasks for about 1 week at 20 °C in darkness and then exposed to near UV light under a 16 h photoperiod for 3 weeks at 15 °C. The mycelium-colonised grain was air dried and stored in a refrigerator at 4 °C until usage.

On the date of inoculation, the grain with *Fusarium* mycelium was suspended in tap water for 2 h and then filtered through cheesecloth to obtain a conidial suspension. The suspensions from each of the three isolates were adjusted to 100,000 spores/mL with the aid of a haemocytometer. Equal volumes of suspension were mixed.

Wheat spikes were sprayed with a spore suspension at anthesis at a rate of 100 mL/m^2^. Inoculations were performed individually on each plot at the beginning of anthesis, and repeated about 3 days later at full anthesis. Inoculations were carried out in the evening, when relative air humidity was increasing.

In the experimental field in Cerekwica, in order to maintain a high humidity necessary to infection of heads, plots were mist irrigated for 3 days after inoculation. In the second location in the Radzików field experiment was conducted without mist irrigation, however, the experimental field was located near the river and this area had high relative air humidity.

Severity of *Fusarium* head blight was evaluated twice, 14 and 21 days post inoculation. The presence of FHB (percentage of heads infected per plot) and percentages of head infection were determined. *Fusarium* head blight index was calculated as the combination of disease severity and disease incidence.

After ripening, 30 randomly selected spikes per plot were harvested. Grain was threshed using laboratory thresher. Percentage of kernels damaged by *Fusarium* (FDK) was determined by dividing samples into two categories: healthy looking kernels (plump, normal color, no visual infection; slightly shrivelled of normal color) and kernels with visible symptoms of *Fusarium* damage (discolored kernels of normal size or slightly shrivelled, pinkish-white, shrivelled kernels = “tombstone”) [70].

The content of the type B trichothecenes (deoxynivalenol [DON], 3-acetyldeoxynivalenol [3AcDON], 15-acetyldeoxynivalenol [15AcDON], nivalenol [NIV]) in triticale and wheat grain was analysed, using the technique of gas chromatography.

Mycotoxins were extracted from 5 g of ground grains using 25 mL of an aqueous solution of acetonitrile (acetonitrile:water 84:16 *v*/*v*) was shaken on the laboratory shaker overnight. Samples were centrifuged (3000 rpm·min^−1^, 5 min), and the extract was purified with MycoSep^®^ 227 Trich+ columns (Romer Labs Inc., Union, MO, USA). One microlitre of the internal standard solution (chloralose) was added to 4 mL of purified extract. The solvent was evaporated to dryness in the air stream. Mycotoxins were derivatised to the trimethylsilyl derivatives using the derivatising agent Sylon BTZ [BSA (N,O-Bis(trimethylsilyl)acetamide):TMCS (Chlorotrimethylsilane):TMSI (N-trimethylsilyimidazole), 3:2:3] (Supelco, Bellefonte, PA, USA). After dissolution of sample in isooctane, excess of derivatising agent was decomposed and removed with water. The organic layer was transferred to autosampler vial and analysed chromatographically with gas chromatograph SRI 8610C (SRI Instruments, Torrance, CA, USA), with BGB-5MS column of 30 m in length, and an internal diameter of 0.25 mm.

Hydrogen was a carrier. Elution was carried out in the temperature gradient. Mycotoxin detection was carried out using electron capture detector (ECD). Identification of individual compounds was made by comparing the retention times of the pure standards of mycotoxins. The concentration of mycotoxins was established on the basis of the calibration curve, using chloralose as the internal standard. The content of type B trichothecenes was expressed as toxin weight (mg) per grain weight (kg).

The content of zearalenone (ZEN) was determined using a quantitative direct competitive enzyme-linked immunosorbent assay (ELISA) AgraQuant^®^ ZON 40/1000 (LOD 10 ppb) (Romer Labs Inc., Union, MO, USA). A 5 g of the ground sample was placed in a conical 50 mL Falcon centrifuge tube and then 25 mL of the solvent (methanol-water 70:30 *v*/*v*) was added. The sample was extracted for 1 h on a shaker and then centrifuged (1620 *g*, 5 min). The obtained extract was analysed with ELISA method according to the procedure described by Romer Labs. The content of zearalenone was expressed as toxin weight (mg) per grain weight (kg).

The statistical analysis was performed using Microsoft^®^ Excel 2010/XLSTAT©-Pro (Version 2015.2.02.18135, Addinsoft, Inc., Brooklyn, NY, USA). Differences between the two cereal species were compared using the Kruskal–Wallis one-way analysis of variance (XLSTAT procedure: *Comparison of k samples—Kruskal-Wallis, Friedman, …*). The Kruskal-Wallis test was selected because most of the variables did not follow normal distribution. Comparison of the variances of triticale and wheat samples (FHBi, FDK and mycotoxin concentrations) were performed using parametric Fisher’s F test (XLSTAT procedure: *Two-sample comparison of variances*). The relationships between FHBi, FDK and mycotoxin concentrations for wheat and triticale were investigated by Pearson correlation tests (XLSTAT procedure: *Correlation tests*). Prior to analysis, data which did not follow normal distribution was log10 transformed to normalise residual distributions. Multivariate data analysis method was applied to the data on FHB resistance. Principal component analysis (XLSTAT procedure: *Principal Component Analysis PCA*) was used to show how the two cereal species are distributed with respect to the main variation described in the first two components and how variables influence the construction of the two components. PCA results also revealed associations among variables measured by the angle between variable vectors.

## Figures and Tables

**Table 1:** Comparison of wheat and triticale in resistance to *Fusarium* head blight and *Fusarium* toxin accumulation in grain.

Cereal	FHB Index (%)	FDK (%)	DON (mg/kg)	3AcDON (mg/kg)	15AcDON (mg/kg)	NIV (mg/kg)	TCT B (mg/kg)	ZEN (mg/kg)
Wheat (*n* = 34)	28.0 b	55.6 b	11.65 a **	2.00 a	1.47 a **	4.13 a *	19.25 a **	0.60 a
Triticale (*n* = 32)	19.2 a	40.2 a	14.12 b **	1.82 a	1.91 b **	5.19 b *	23.03 b **	0.66 a

Values within the same column followed by the different letters are significantly different at the level of probability < 0.001, ** < 0.01 or * < 0.05. FHB: *Fusarium* head blight; FDK: *Fusarium* damaged kernels; DON: deoxynivalenol; 3AcDON: 3-acetyldeoxynivalenol; 15AcDON: 15-acetyldeoxynivalenol; NIV: nivalenol; TCT B: type B trichothecenes; ZEN: zearalenone.

**Table 2:** Coefficients of correlations between FHB (*Fusarium* head blight) index, *Fusarium* damaged kernels (FDK), and type B trichothecenes (DON, 3AcDON, 15AcDON, NIV, TCT B) and zearalenone (ZEN) accumulation in grain of 32 triticale lines (below, diagonal), and 34 wheat lines (above, diagonal) from experiments in Cerekwica and Radzików.

Variables	FHB Index	FDK	DON	3AcDON	15AcDON	NIV	TCT B ^a^	ZEN
FHB index	-	0.604 ***	0.538 ***	0.630 ***	0.333 ^ns^	0.326 ^ns^	0.576 ***	0.412 *
FDK	0.408 ^b,^*	-	0.451 **	0.529 ***	0.336 *	0.314 ^ns^	0.504 **	0.233 ^ns^
DON	0.398 *	0.214 ^ns^	-	0.845 ***	0.889 ***	0.251 ^ns^	- ^c^	-0.014 ^ns^
3AcDON	0.536 **	0.347 ^ns^	0.885 ***	-	0.736 ***	0.262 ^ns^	-	0.170 ^ns^
15AcDON	0.267 ^ns^	0.232 ^ns^	0.890 ***	0.804 ***	-	0.057 ^ns^	-	-0.165 ^ns^
NIV	0.180 ^ns^	0.465 **	-0.406 *	-0.262 ^ns^	-0.339 ^ns^	-	-	0.406 *
TCT B ^a^	0.357 *	0.451 **	-	-	-	-	-	0.115 ^ns^
ZEN	0.166 ^ns^	0.490 **	0.350 *	0.472 **	0.285 ^ns^	0.295 ^ns^	0.501 **	-

^a^ Sum of DON, 3AcDON, 15AcDON, and NIV; ^b ns^ not significant. *, **, and *** correlation coefficient is significant at *p* < 0.05, 0.01, and 0.001, respectively; ^c^ Coefficients of correlation between TCT B and individual trichothecenes were not included in the table.

## Supplementary Materials

The following are available online at www.mdpi.com/2072-6651/8/10/301/s1. Figure S1: Daily air temperature and sum of precipitation in Cerekwica (C) and Radzików (R) during *Fusarium* inoculation of triticale and wheat heads. Table S1: Flowering periods of winter triticale and winter wheat in 2012 in two experimental locations. Table S2: Average daily air temperature and sum of precipitation in Cerekwica and Radzików from triticale and wheat flowering until the end of harvest in 2012. Table S3: Resistance to *Fusarium* head blight and mycotoxin accumulation in grain of 29 lines and three cultivars of winter triticale inoculated with *Fusarium culmorum* isolates in the field experiments in Radzików and Cerekwica. FHB: *Fusarium* head blight; FDK: *Fusarium* damaged kernels; DON: deoxynivalenol; 3AcDON: 3-acetyldeoxynivalenol; 15AcDON: 15-acetyldeoxynivalenol; NIV: nivalenol; TCT B: type B trichothecenes; ZEN: zearalenone. Table S4: Resistance to *Fusarium* head blight and mycotoxin accumulation in grain of 32 lines and two cultivars of winter wheat inoculated with *Fusarium culmorum* isolates in the field experiments in Radzików and Cerekwica. FHB: *Fusarium* head blight; FDK: *Fusarium* damaged kernels; DON: deoxynivalenol; 3AcDON: 3-acetyldeoxynivalenol; 15AcDON: 15-acetyldeoxynivalenol; NIV: nivalenol; TCT B: type B trichothecenes; ZEN: zearalenone.
