# Supplementary material for: Higher Fusarium Toxin Accumulation in Grain of Winter Triticale Lines Inoculated with Fusarium culmorum as Compared with Wheat
Source: Toxins (Basel). 2016 Oct 18;8(10):301. doi: 10.3390/toxins8100301 (PMC5086661; doi:10.3390/toxins8100301)
Supplement: Supplementary file 1 [file toxins-08-00301-s001.pdf]

Supplementary Materials: Higher *Fusarium* Toxins Accumulation in Grain of Winter Triticale Lines Inoculated with *Fusarium culmorum* as Compared with Wheat

Tomasz Góral, Halina Wiśniewska, Piotr Ochodzki and Dorota Walentyn-Góral

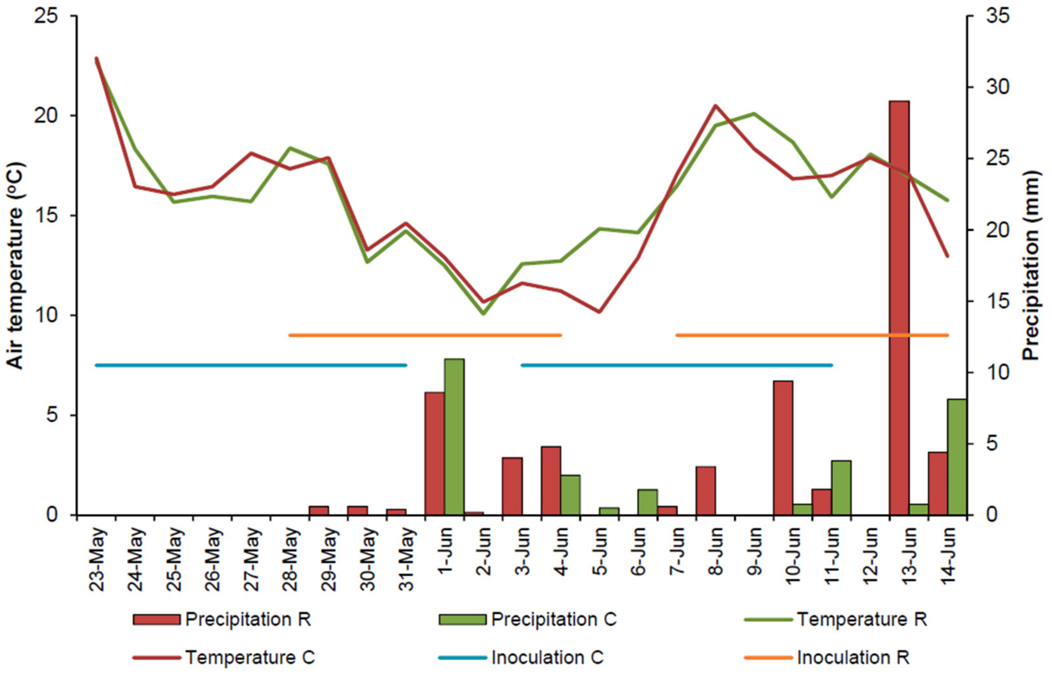

Figure S1. Daily air temperature and sum of precipitation in Cerekwica (C) and Radzików (R) during *Fusarium* inoculation of triticale and wheat heads.

Table S1. Flowering periods of winter triticale and winter wheat in 2012 in two experimental locations.

Location	Triticale	Wheat
Cerekwica	23–30 May	3 May–11 June
Radzików	28 May–4 June	7–14 June

Table S2. Average daily air temperature and sum of precipitation in Cerekwica and Radzików from triticale and wheat flowering until the end of harvest in 2012.

Days/month	Cerekwica		Radzików	
	Temperature (°C)	Precipitation (mm)	Temperature (°C)	Precipitation (mm)
22–31 May	17.0	0	16.8	1.6
1–30 June	16.6	112.3	17.4	92.6
1–31 July	19.8	152.1	21.0	79.6
1–15 August	18.5	26.7	18.8	38.8

Table S3. Resistance to *Fusarium* head blight and mycotoxin accumulation in grain of 29 lines and three cultivars of winter triticale inoculated with *Fusarium culmorum* isolates in the field experiments in Radzików and Cerekwica. FHB: *Fusarium* head blight; FDK: *Fusarium* damaged kernels; DON: deoxynivalenol; 3AcDON: 3-acetyldeoxynivalenol; 15AcDON: 15-acetyldeoxynivalenol; NIV: nivalenol; TCT B: type B trichothecenes; ZEN: zearalenone.

No.	Line/Cultivar	FHB Index (%)	FDK (%)	DON (mg/kg)	3AcDON (mg/kg)	15AcDON (mg/kg)	NIV (mg/kg)	TCT B [b] (mg/kg)	ZEN (mg/kg)
1	DL 446/08 [a]	9.7	20.2	14.04	1.18	2.01	2.80	20.02	0.52
2	DS.9	11.1	29.2	14.76	1.64	2.44	2.16	21.00	0.36
3	DD 436/07	11.8	43.7	14.91	1.87	2.04	7.80	26.62	0.77
4	Borwo cv.	12.1	49.4	15.22	2.37	1.55	10.17	29.31	1.23
5	BOHD 826-3	12.3	45.6	6.25	0.77	1.03	6.97	15.02	0.43
6	BOHD 898-1	12.9	33.9	5.88	0.62	0.86	6.06	13.42	0.39
7	MAH 31011-2	14.5	33.8	6.14	0.58	0.64	5.23	12.59	0.28
8	LD 121/08	14.5	31.0	11.45	1.49	1.82	7.89	22.65	0.41
9	DS 1238	14.7	38.3	15.09	1.61	2.10	2.90	21.69	0.58
10	DD 466/07	14.8	47.2	12.20	1.78	1.87	6.86	22.70	1.61
11	BOHD 1049-1	15.1	43.8	9.69	1.17	1.61	5.79	18.26	0.60
12	MAH 33097-1	15.5	40.9	9.62	1.22	1.38	8.81	21.03	0.53
13	Mikado cv.	16.9	34.7	13.67	1.16	1.13	5.14	21.09	0.46
14	DAST 27/10	17.3	33.9	15.16	1.76	1.60	2.90	21.41	0.41
15	BOH 623-1	18.2	39.0	14.98	2.42	2.24	3.53	23.17	0.34
16	BOHD 1190-1	18.9	41.2	9.54	1.03	1.32	7.73	19.62	0.80
17	DC 06080-56	19.0	39.0	17.72	2.51	2.48	4.19	26.90	0.66
18	BOH 537-2	19.8	34.7	13.40	1.78	1.88	4.36	21.42	0.55
19	CM 9/10	20.0	51.9	16.88	2.02	2.36	5.94	27.20	1.20
20	MAH 33544-3	21.7	40.0	15.99	2.32	2.43	3.06	23.81	0.59
21	DL 593/07	21.7	40.8	16.42	1.90	2.61	3.54	24.46	0.34
22	MAH 32247-1	21.8	38.9	15.75	1.78	2.19	4.86	24.58	0.70
23	DC 03326/06/2	21.9	39.4	15.31	2.24	1.93	3.03	22.51	0.86
24	MAH 31938-4	22.7	43.7	10.52	1.61	1.59	6.56	20.28	0.48
25	MAH 33405-1	22.8	48.6	15.55	2.26	2.23	7.10	27.13	0.87
26	DC 04294/04/1	23.4	50.2	25.87	2.88	2.96	3.89	35.60	0.98
27	DS 1260	24.3	38.5	15.17	2.17	1.87	3.19	22.40	0.60
28	DC 04294/04/2	25.3	43.7	22.40	2.62	3.14	2.85	31.01	0.95
29	MAH 33881-1/3	26.3	42.8	23.13	3.40	3.25	4.79	34.57	0.57
30	BOHD 1233-2	27.8	54.7	14.16	1.93	2.13	8.69	26.91	0.70
31	MAH 33767-3	30.6	33.4	9.92	2.22	0.98	4.32	17.44	1.06
32	Fredro cv.	36.0	41.7	14.95	1.78	1.60	2.88	21.20	0.36
	**Mean**	**19.2**	**40.2**	**14.12**	**1.82**	**1.91**	**5.19**	**23.03**	**0.66**
	**$LSD_{0.05}$**	**13.0**	**15.2**	**9.92**	**1.60**	**1.71**	**6.10**	**10.02**	**0.62**

[a] Lines ordered according to FHB index; [b] sum of DON, 3AcDON, 15AcDON, and NIV.

Table S4. Resistance to *Fusarium* head blight and mycotoxin accumulation in grain of 32 lines and two cultivars of winter wheat inoculated with *Fusarium culmorum* isolates in the field experiments in Radzików and Cerekwica. FHB: *Fusarium* head blight; FDK: *Fusarium* damaged kernels; DON: deoxynivalenol; 3AcDON: 3-acetyldeoxynivalenol; 15AcDON: 15-acetyldeoxynivalenol; NIV: nivalenol; TCT B: type B trichothecenes; ZEN: zearalenone.

No.	Line/Cultivar	FHB Index (%)	FDK (%)	DON (mg/kg)	3AcDON (mg/kg)	15AcDON (mg/kg)	NIV (mg/kg)	TCT B [b] (mg/kg)	ZEN (mg/kg)
1	STH 9059 [a]	17.4	34.4	6.95	0.85	0.72	2.86	11.37	0.37
2	AND 340/06-1	18.4	61.3	9.07	1.42	1.27	3.61	15.37	0.40
3	POB 0111	19.5	50.5	10.19	1.62	1.55	3.25	16.60	0.25
4	POB 262/07	20.2	50.1	6.57	1.18	0.81	5.01	13.57	0.85
5	KBP 10 40	21.1	45.4	8.67	1.19	1.35	3.17	14.38	0.23
6	NAD 06133	21.4	44.3	10.43	1.72	1.45	3.03	16.63	0.46
7	POB 0911	21.7	42.0	13.57	1.14	2.04	3.99	20.74	0.42
8	SMH 8670	22.8	56.2	11.66	1.76	1.42	4.02	18.85	0.33
9	POB 0211	23.0	55.0	14.05	2.59	2.13	1.88	20.64	0.21
10	AND 4015/09	24.1	60.1	8.80	1.64	0.95	4.27	15.65	0.54
11	DCh 4763/07	25.9	55.2	10.70	1.75	1.05	5.20	18.70	0.93
12	DM 2728/09	26.0	44.9	12.26	2.89	1.89	6.60	23.64	0.32
13	NAD 08161	26.3	51.0	10.27	1.56	1.34	3.69	16.85	0.46
14	POB 457/07	26.4	58.6	7.21	0.96	0.93	3.39	12.48	0.30
15	STH 102	26.8	66.1	13.94	1.58	1.63	3.28	20.42	0.23
16	DL 528/08	26.8	44.2	12.06	2.16	1.30	3.37	18.88	0.55
17	POB 1011	27.2	58.1	15.41	2.58	2.00	3.82	23.82	0.29
18	KBP 08 20	27.5	45.3	9.17	1.33	0.89	4.66	16.05	0.55
19	STH 0290	27.9	58.8	8.70	1.91	0.97	4.10	15.67	1.10
20	SMH 8671	28.0	56.9	12.43	2.38	1.62	4.32	20.75	0.44
21	DM 3313/09	28.1	52.5	6.80	1.13	1.03	3.10	12.06	0.89
22	DC 704/08-4	28.2	64.6	13.62	2.68	1.81	6.26	24.37	0.56
23	DD 343/07	28.3	62.3	9.51	1.53	1.18	5.02	17.23	1.17
24	STH 087	29.2	47.8	10.64	2.08	1.52	2.51	16.76	1.20
25	KBP 10 2	29.3	57.7	11.58	2.28	1.40	2.95	18.21	0.51
26	Tonacja cv.	29.6	58.0	14.91	2.69	1.73	4.38	23.71	0.48
27	NAD 06130	30.3	63.3	14.90	2.61	2.26	2.62	22.39	0.48
28	HRSM 789	31.7	69.6	19.68	3.39	2.24	10.07	35.39	1.65
29	STH 104	32.2	53.0	12.87	2.50	1.66	2.53	19.55	0.57
30	KBP 09 22	33.5	69.2	10.97	2.09	1.37	3.75	18.18	0.58
31	KWS Ozon cv.	35.0	61.0	14.35	2.40	1.70	3.94	22.39	0.52
32	DD 414/07-4	39.2	62.1	14.64	2.90	1.74	3.89	23.17	1.41
33	DC 44/08-4 (S)	47.8	65.2	12.01	2.16	1.29	5.72	21.18	0.88
34	KBP 09 20 (S)	50.1	67.1	17.51	3.31	1.83	6.08	28.72	0.42
	**Mean**	**28.0**	**55.6**	**11.65**	**2.00**	**1.47**	**4.13**	**19.25**	**0.60**
	**$LSD_{0.05}$**	**19.6**	**13.9**	**7.88**	**1.50**	**1.11**	**4.09**	**9.43**	**0.90**

[a] Lines ordered according to FHB index; [b] sum of DON, 3AcDON, 15AcDON, and NIV.
